# Supplementary material for: The Impact of the Injected Mass of the Gastrin-Releasing Peptide Receptor Antagonist on Uptake in Breast Cancer: Lessons from a Phase I Trial of [99mTc]Tc-DB8
Source: Pharmaceutics. 2025 Jul 31;17(8):1000. doi: 10.3390/pharmaceutics17081000 (PMC12389326; doi:10.3390/pharmaceutics17081000)

Primary Data:
Supplementry Table S1. Tumor Uptake (SUV_{max}), ER-positive primary tumors.

Time point (h pi)	Injected DB8 mass (µg)	Individual values				
2	40	1.96	1.89	2.06	2.55	1.72
	80	5.08	5.76	6.5	3.28	5.9
	120	2.15	5.7	2.58	2.97	2.72
4	40	1.34	1.14	1.12	1.27	1.02
	80	3.67	4.55	6.49	2.36	6.88
	120 g	1.7	5.75	2	2.26	2.19
6	40	0.99	1.09	1.03	1.08	0.67
	80	3.13	4.71	5.01	1.55	5.42
	120	0.93	4.94	1.16	2.11	2.03

Supplementary Table S2. Tumor-to-contralateral site ratios, ER-positive tumors

Time point (h pi)	Injected DB8 mass (µg)	Individual values				
2	40	2.55	3.94	7.63	6.22	2.82
	80	9.07	9	12.04	9.94	15.95
	120	2.95	4.52	2.24	2.45	2.92
4	40	3.12	8.14	6.59	5.29	4.08
	80	11.47	13.38	7.13	3.28	25.48
	120 g	3.21	4.39	5.26	2.9	2.43
6	40	2.2	10.9	11.44	5.68	1.97
	80	13.04	31.4	3.63	5.54	31.88
	120	3.88	4.15	3.52	3.63	5.63

Supplementary Table S3. Tumor-to-liver ratios, ER-positive tumors

Time point (h pi)	Injected DB8 mass (µg)	Individual values				
2	40	1.22	1.05	1.87	1.53	1.04
	80	2.62	2.76	2.23	1.52	2.94
	120	1.65	3.99	1.88	1.83	1.84
4	40	0.93	1.06	0.73	0.86	0.8
	80	3.06	3.5	2.17	1.46	3.07
	120 g	1.42	2.71	1.5	2.53	2.06
6	40	0.71	0.78	1.04	0.86	1.22
	80	2.68	4.03	2.04	1.25	2.54
	120	0.95	3.71	1.22	1.7	2.7

Supplementary Table S4. Tumor uptake (SUV_{max}) in primary ER-positive and ER-negative tumors

Estrogen receptor status	Individual values				
ER-	2.97	2.39	1.68	1.11	
ER+	5.08	5.76	6.5	3.28	5.9

Metadata:
Table S1. Presents original SUVmax values for every patient with estrogen receptor (ER)-positive tumors. The values were provided by Siemens Symbia Intevo Bold scanner software after the reconstruction of SPECT/CT images.
Table S2. Presents tumor-to-contralateral site ratios for patients with ER-positive primary tumors. To obtain the data, the region of interest (VOI) placed over the tumor was copied on the contralateral breast and the ratio of SUVmax values was recorded.
Table S3. Presents tumor-to-liver ratios for patients with ER-positive primary tumors. To obtain the data, the region of interest (VOI) placed over the tumor was copied on the liver and the ratio of SUVmax values was recorded.
Table S4. Presents tumor uptake (SUVmax) in primary ER-positive and ER-negative tumors, 2 h after injection of [^{99m}Tc]Tc-DB8. The values were provided by Siemens Symbia Intevo Bold scanner software after the reconstruction of SPECT/CT images.

Code or Scripts: Differences between uptakes and tumor-to-organ ratios at different injected masses of the DB8 were analyzed using one-way Analysis of Variance (ANOVA). 2-sided P value of less than 0.05 was considered significant. Paired t-tests were used to analyze differences between uptakes in organs at different time points. The statistical analysis was performed using Prism 9 for Windows (Graph Pad Software, LLC. Boston, MA).

Differences between tumor uptakes between patients with ER-positive and ER-negative primary tumors were analyzed using the nonparametric Mann–Whitney U test. 2-sided P value of less than 0.05 was considered significant. Paired t-tests were used to analyze differences between uptakes in organs at different time points. The statistical analysis was performed using Prism 9 for Windows (Graph Pad Software, LLC. Boston, MA).

Supplementary Figure S1. Body mass of patients included in the study. There was no significant difference ($p > 0.05$, one-way ANOVA test) between groups.

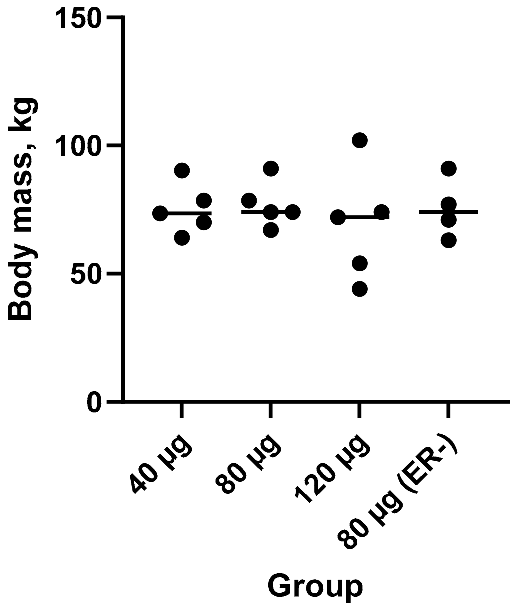

Supplementary Figure S2. Representative SPECT/CT images (Patients 2, 6 and 15) of primary ER-positive tumors 2 h after injection of [^{99m}Tc]Tc-DB8 with 40, 80 and 120 µg DB8. Arrows point at tumors. The upper setting of a linear intensity scale is adjusted to SUV 2 in all images.

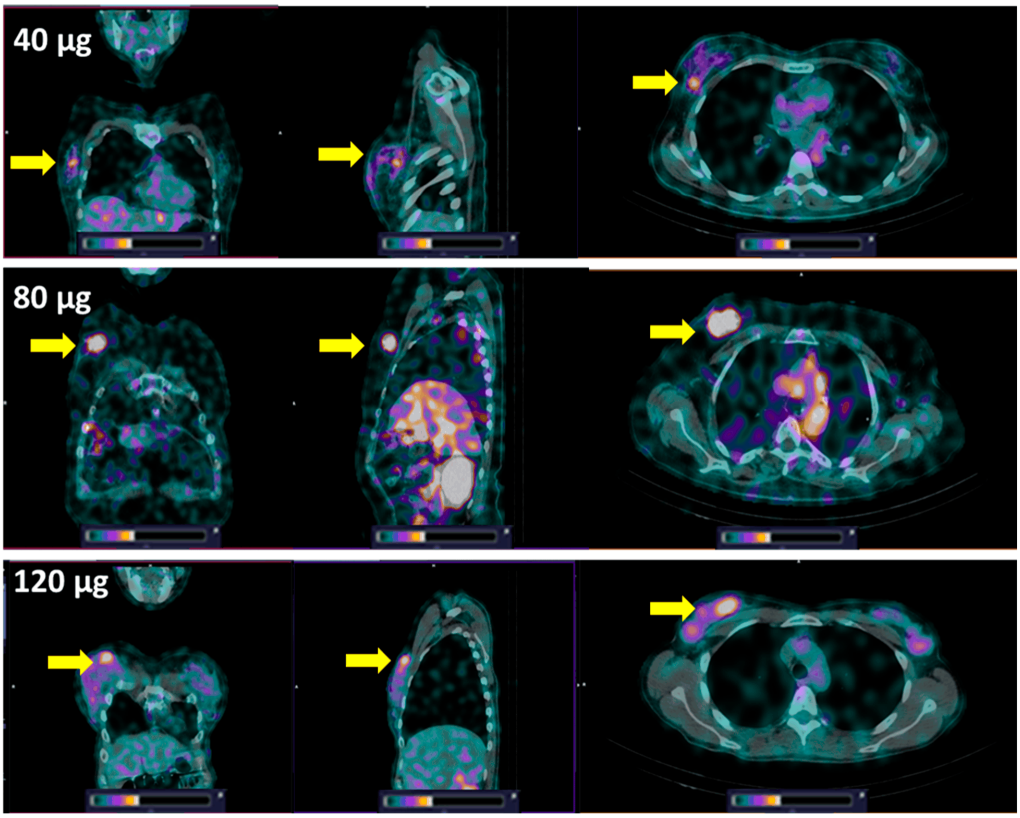

Supplementary Figure S3. Statistical analysis of the tumor uptake (SUV) at different time points after injection of 80 µg [^{99m}Tc]Tc-DB8. Paired t-test was used because the uptake at different time points was measured in the same patients.

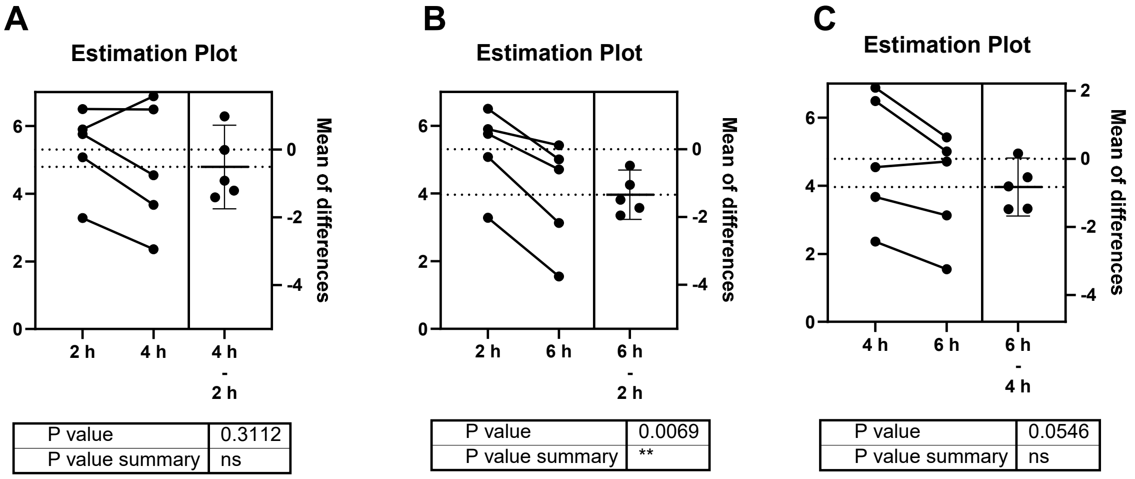

Supplementary Figure S4. Uptake (SUVmax) in primary ER-positive breast cancer lesions 2 h after injection of [^{99m}Tc]Tc-DB8 as a function of injected mass per kg of body weight.

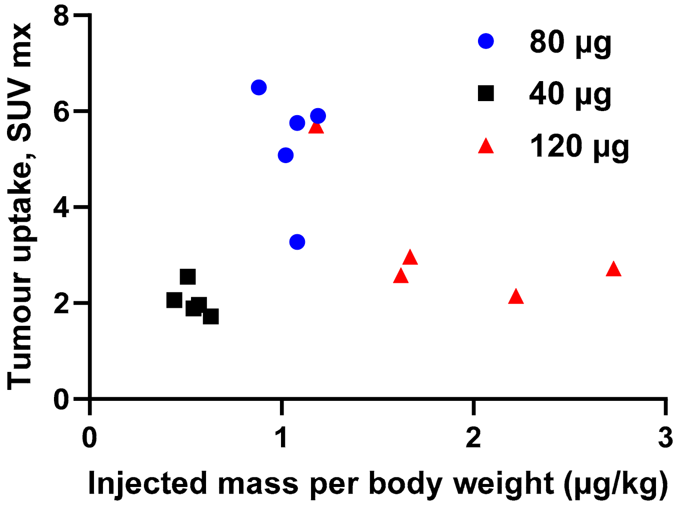

Supplement: Supplementary file 1 [file pharmaceutics-17-01000-s001.zip › pharmaceutics-3721760-supplementary.pdf]
